# Neurodevelopment Outcomes in Very-Low-Birth-Weight Infants with Metabolic Bone Disease at 2 Years of Age

**DOI:** 10.3390/children11010076

**Published:** 2024-01-09

**Authors:** Yu-Wen Chen, Yu-Jun Chang, Lih-Ju Chen, Cheng-Han Lee, Chien-Chou Hsiao, Jia-Yuh Chen, Hsiao-Neng Chen

**Affiliations:** 1 Department of Neonatology, Changhua Christian Children’s Hospital, No. 320, Xuguang Road, Changhua City 500010, Taiwan; 2 Big Data Center, Changhua Christian Hospital, No. 135, Nanxiao Street, Changhua City 500209, Taiwan; 3 Department of Post-Baccalaureate Medicine, College of Medicine, National Chung Hsing University, No. 145, Xingda Road, South District, Taichung City 402202, Taiwan; 4 School of Medicine, Chung-Sun Medical University, No. 110, Sec. 1, Jianguo N. Road, South District, Taichung City 402306, Taiwan

**Keywords:** metabolic bone disease, neurodevelopmental outcomes, very-low-birth-weight infants, risk factors, Bayley Scales of Infant Development-III

## Abstract

Metabolic bone disease (MBD) predominantly affects preterm infants, particularly very-low-birth-weight (VLBW) infants weighing <1500 g. However, there are limited reports on MBD and neurodevelopmental outcomes. This study aimed to analyze the risk factors for MBD and understand its impact on neurodevelopmental outcomes at 2 years of corrected age. Overall, 749 VLBW infants weighing <1350 g at birth were enrolled. Exclusion criteria were major congenital abnormalities, chromosomal abnormalities, and loss of follow-up on the Bayley Scales of Infant Development, Third Edition (BSID-III) test at 24 months of corrected age. Infants were retrospectively assessed by a trained case manager using the BSID-III test at 6, 12, and 24 months old. Infants were categorized as with or without MBD according to radiographic signs. Of those enrolled, 97 VLBW infants were diagnosed with MBD, compared to 362 VLBW infants without MBD. The proportion of infants that completed three follow-ups was 86%. At the assessment at 2 years of age, infants with MBD had lower and more significant differences in motor, language, and cognitive composites. MBD is associated with poor neurodevelopmental outcomes in cognitive, motor, and language composites for VLBW infants at 24 months of corrected age.

## 1. Introduction

Metabolic bone disease (MBD) is a metabolic disorder that primarily affects preterm infants, particularly very-low-birth-weight (VLBW) infants weighing less than 1500 g at birth [1,2,3]. This usually occurs in newborns less than 28 weeks of gestation, with approximately 10% of premature infants experiencing a fracture upon reaching a corrected gestational age of 36 to 40 weeks [4,5]. MBD often occurs in preterm infants 6–8 weeks after birth [4]. The pathogenesis of MBD remains unclear and is likely to be multifactorial [6,7]. It has been established that MBD is associated with rickets and fractures, low peak bone mass, and poorer respiratory outcomes [1,7].

However, there are limited reports on MBD with regard to neurodevelopmental outcomes. Therefore, the aim of this study is to analyze the risk factors for MBD in VLBW infants and understand the impact of MBD on neurodevelopmental outcomes at 2 years of corrected age.

## 2. Materials and Methods

This is a retrospective, single-center cohort study that included preterm VLBW infants weighing less than 1350 g admitted to our children hospital’s neonatal intensive care unit (NICU) between 2011 and 2019. This study was conducted according to the principles of the Declaration of Helsinki and was approved by the Research Ethics Board Committee of Changhua Christian Children’s Hospital (CCH IRB No. 220531; approval date: 22 June 2022). The requirement for informed consent was waived owing to the retrospective nature of this study.

### 2.1. Study Population

VLBW infants weighing less than 1350 g admitted to the children’s hospital between January 2011 and December 2019 were enrolled. Infants who had major congenital or chromosomal abnormalities or were lost to follow-up during the Bayley Scales of Infant Development, Third Edition (BSID-III) test at 24 months of corrected age were excluded.

### 2.2. Strategy of Nutrition Management

Within our institution, the order of preference for enteral nutrition is to use breastmilk as the primary option, followed by human milk from the milk bank as a secondary option, and preterm infant formula as a fallback option when neither of the first two options are feasible. For parenteral nutrition (PN), we administer calcium gluconate and organic phosphorus, targeting 60–80 mg/kg/day of calcium and 45–60 mg/kg/day of phosphorus during the growth phase, with a weekly monitoring of calcium (Ca), phosphorus (P), and magnesium (Mg) levels for adjustments. Upon reaching a daily fluid intake of 100 mL/kg/day, total parenteral nutrition (TPN) is discontinued, and Human Milk Fortifier (HMF) is added to breastmilk. This ensures that breastmilk contains sufficient minerals, micronutrients, and proteins to meet the nutritional needs of growing preterm infants. The study used Enfamil HMF (providing 1 g of protein, 90 mg of calcium, and 50 mg of phosphorus per 4-pack) and Similac HMF (providing 1 g of protein, 118 mg of calcium, and 66 mg of phosphorus per 4-pack), alternating monthly. Breastmilk additives begin with 2 packets in 100 mL of breastmilk on the first three days and 4 packets in 100 mL of breastmilk from the fourth day onwards. Since 2017, we have integrated vitamin D supplementation into the nutritional management of preterm infants. Vitamin D supplementation commences approximately one week after birth, following the achievement of target Ca/P supplementation in PN and stabilization of oral feeding post the early trophic feeding period. The initial dose is 400 IU/day using LiquiD P&B (Brand: ULONG Pharmaceutical, Taipei, Taiwan). In cases of elevated and rising alkaline phosphatase (ALP) levels (>450 U/L), the vitamin D3 supplement is adjusted upward by 400 IU/day for each increment, up to a maximum of 1200 IU/day.

### 2.3. Patient Demographic Data

Demographic data included maternal age; obstetrical conditions, such as preeclampsia or preterm premature membrane rupture; use of antenatal glucocorticoids; type of delivery; multiple births; gestational age; birth weight; small for gestational age status (defined as birth weight for gestational age <10th percentile); sex, Apgar score at 5 min after birth; support during fetal–neonatal transition; respiratory distress syndrome (RDS); surfactant replacement; duration of invasive respiratory support in the NICU; culture-proven sepsis; necrotizing enterocolitis (NEC) stage ≥ 2 [8]; bronchopulmonary dysplasia (BPD, defined as needing supplemental oxygen for a cumulative duration of >28 days); postnatal steroids for BPD; patent ductus arteriosus (PDA) stage requiring operation; retinopathy of prematurity (ROP) ≥ stage 3; intraventricular hemorrhage (IVH) ≥ grade 3; periventricular leukomalacia (PVL); use of vitamin D3; duration of PN used (reaching an enteral feeding volume of 100 mL/kg/day); and peak serum ALP levels.

### 2.4. Definitions

Currently, there is no consensus regarding the gold standard for an early diagnosis of MBD [9]. Traditionally, the measurement of serum ALP, serum calcium, and serum phosphate is used for screening [10]. In previous reviews, the cut-off level of serum ALP for diagnosing MBD has remained inconclusive [11,12]. We used radiography for the diagnosis of MBD, which highlighted radiographic signs such as the presence of bone rarefaction associated with metaphyseal alterations or subperiosteal bone formations associated with the presence of spontaneous fractures [1]. Images were evaluated by one attending physician.

### 2.5. Neurodevelopmental Outcome Assessment

Infants were assessed by a trained case manager using the BSID-III test at 6, 12, and 24 months old. For these assessments, data were collected retrospectively.

### 2.6. Statistical Methods

Continuous variables, such as gestational age, body weight at birth, Apgar score at 5 min after birth, BSID-III scales, length of PN used, and peak serum ALP levels are described as medians and interquartile ranges (IQR, 25–75th percentile). Categorical variables are described as numbers and percentages. In this study, we divided the preterm infants into two groups according to the results of their MBD diagnosis. First, we explored the potential factors associated with MBD. We compared the demographic data of the study groups using the Mann–Whitney U test for continuous data and the chi-squared test or Fisher’s exact test for categorical data.

Next, we evaluated the changes in the BSID-III scores of each patient over time. We conducted single-variable analyses of all potential correlates to identify the interfering factors for multiple regression analyses. Variables with significant correlation (*p* < 0.05) in the single-variable analysis were included in the multivariate regression model. Only those factors with a significant correlation (*p* < 0.05) were retained. After adjusting for other potential risk factors and inter-correlations of data collected from the same infant, multiple generalized linear models with log link and gamma distribution in the generalized estimating equation (GEE) method were used to compare the longitudinal changes in neurodevelopmental impairment (lower BSID-III scores) between the no MBD and MBD groups. All statistical analyses were conducted using the SPSS software for Windows, version 22.0 (IBM Corp., Armonk, NY, USA). *p*-values < 0.05 were considered statistically significant.

## 3. Results

**Figure 1:**
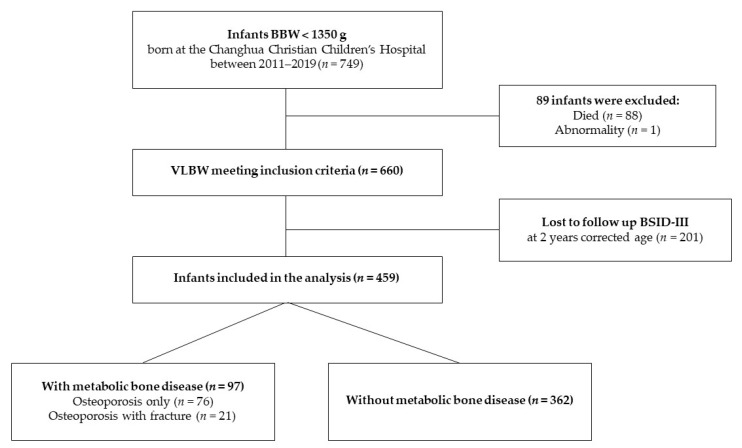
Flowchart illustrating the patient enrollment process in the study. BBW, birth body weight; VLBW, very low birth weight.

During the study period, 953 consecutive infants with VLBW were admitted to our NICU. Among these, 749 infants with a birth weight below 1350 g were included and enrolled in this study. Of these infants, one was excluded due to major congenital abnormalities, and eighty-eight (11.6%) died during hospitalization (Figure 1). Infant and maternal characteristics are summarized in Table 1. Of the 749 patients, 97 were diagnosed with MBD, 21 of which were associated with fractures. Compared to infants without MBD, the MBD group had a lower gestational age, body weight at birth, and median Apgar score at 5 min after birth; a higher risk of invasive respiratory support during fetal–neonatal transition, RDS, surfactant administration, surgery for PDA, and postnatal use of steroids for BPD; a longer median duration of using PN; and higher median peak serum ALP levels. Regarding maternal characteristics, singletons and babies born via vaginal delivery had a higher risk of MBD.

The comparisons of morbidities between infants with and without MBD are summarized in Table 2. The MBD group had a higher risk of cholestasis, BPD, NEC stage ≥ II, culture-proven sepsis, severe ROP, severe IVH, cystic PVL, and post-hemorrhagic hydrocephalus.

**Figure 2:**
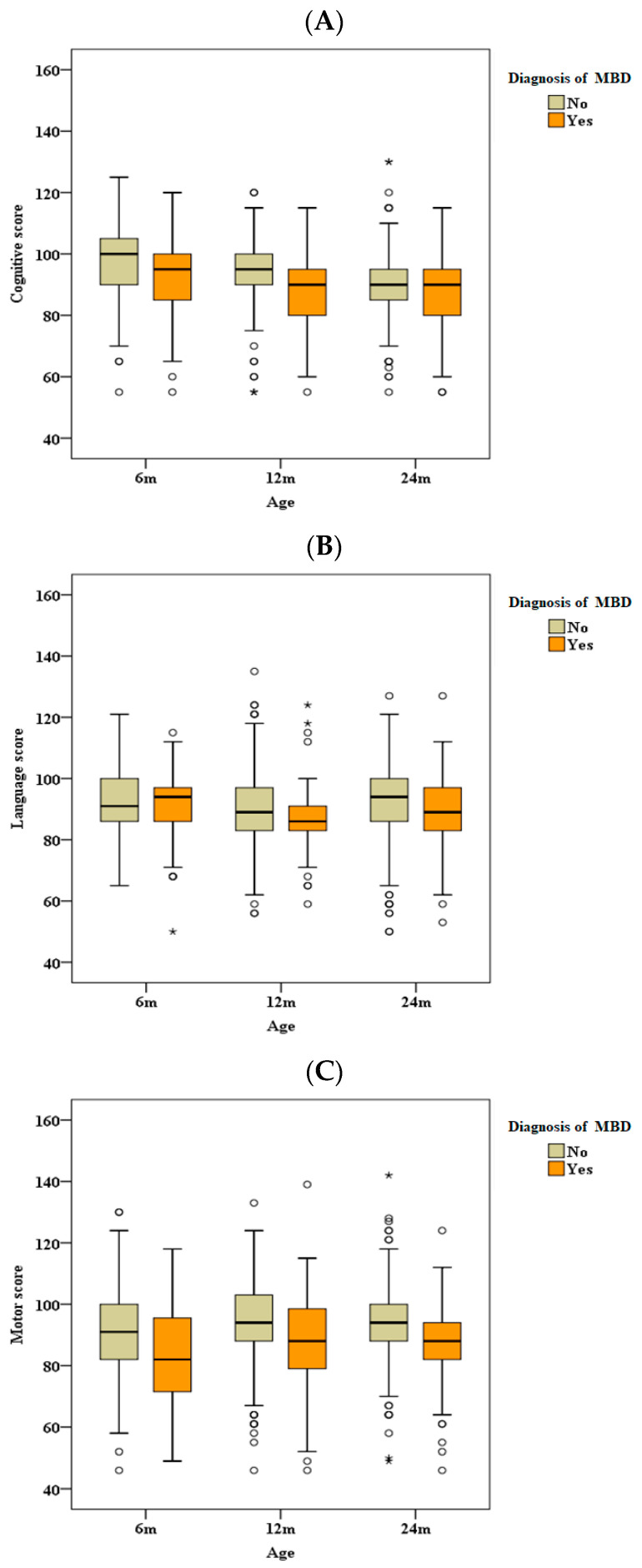
Bayley Scales in infants with or without metabolic bone disease during long-term follow-up. (**A**) Cognitive score, (**B**) language score, and (**C**) motor score. °, values outside the range of 1.5 IQR to 3 IQR, called mild outliers; *, values that are outside the range of more than 3 IQR and are called extreme outliers. MBD, metabolic bone disease; m, months.

The BSID-III score at 2 years of age was compared separately in infants with and without MBD, and the results are summarized in Table 3 and Figure 2. Regarding cognitive outcomes, though both groups had the same median and IQR (median (IQR): 90.0 (85.0–95.0)), the MBD group had a lower maximum scale (minimum and maximum score in the no MBD group was 55 and 130, while in the MBD group it was 55 and 115), indicating that the upper limit of the score is lower in the group with MBD. The motor and language composite scores had similar values at the 2-year evaluation: both were significantly higher in the no MBD group than those in the MBD group (median (IQR): 94.0 (88.0–100.0) vs. 88.0 (82.0–94.0) for the motor composite, *p* < 0.001; 94.0 (86.0–100.0) vs. 90.0 (86.0–97.0) for the language composite, *p* = 0.013).

To investigate whether the severity of MBD has differential effects on neurodevelopmental prognosis, we subgrouped the infants diagnosed with MBD into those with or without fractures to analyze them further. The results showed no significant difference between the two subgroups (Table 4).

To compare the differences in the BSID-III scores between infants with and without MBD and their changes over time, we used the multiple generalized linear models with log link and gamma distribution in the GEE method to analyze and control for potential confounding factors (such as gestational age, cystic PVL, steroids for BPD, intubation during fetal–neonatal transition, and cholestasis). The results showed that the MBD group had significantly lower motor and cognitive composite scores on the BSID-III; however, there was no significant difference in the language composite scores (Table 5).

**Figure 3:**
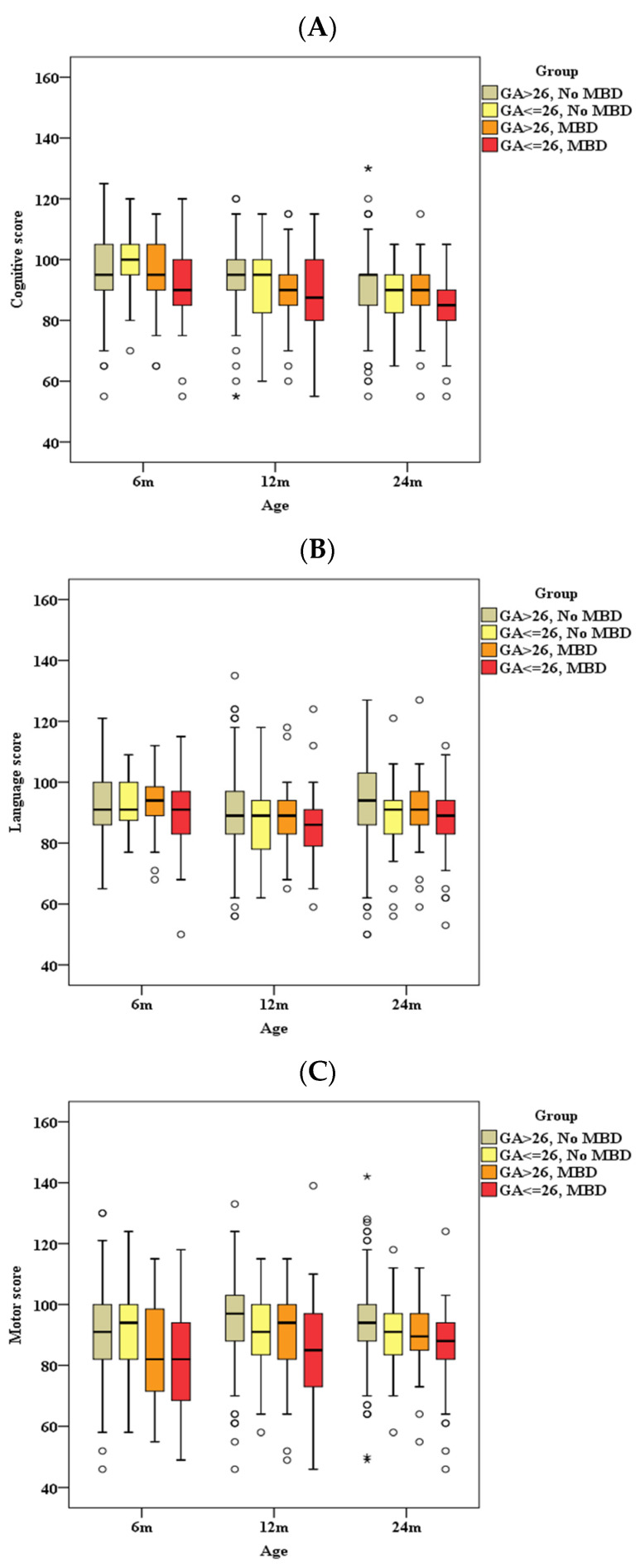
Bayley Scales in a cross-comparison of the four groups (GA ˃ or ≤26 weeks and with/without MBD). (**A**) Cognitive score, (**B**) language score, and (**C**) motor score. °, values outside the range of 1.5 IQR to 3 IQR, called mild outliers; *, values that are outside the range of more than 3 IQR and are called extreme outliers. GA, gestational age; MBD, metabolic bone disease; m, months.

We conducted an ROC curve analysis and observed an increased likelihood of metabolic bone disease in infants displaying a peak ALP level ≥ 500 IU/L, a birth weight less than 850 g, and a gestational period of ≤26 weeks. Subsequently, in order to delineate more accurately the individual influences of prematurity and metabolic bone diseases on neurodevelopmental outcomes, we carried out an additional comparative analysis among four distinct groups (GA ˃ or ≤ 26 weeks and with/without MBD), as illustrated in Figure 3. Our analysis distinctly indicated significant differences in performance across various Bayley-III scores among these four groups (*p* < 0.001). The group GA ˃ 26 weeks and no MBD exhibited the highest scores, followed by GA ≤ 26 weeks and no MBD, and GA > 26 weeks with MBD groups. Conversely, the poorest performance was observed in the GA ≤ 26 weeks with MBD group. This indicates that if patients have GA ≤ 26 weeks, their Bayley-III scores are indeed lower, but if they also have MBD, the scores tend to be worse. Hence, MBD emerges as a noteworthy risk factor influencing neurodevelopmental outcomes.

## 4. Discussion

The comprehensive diagnosis of MBD primarily relies on features such as decreased serum calcium and phosphorus levels, elevated serum ALP and parathyroid hormone (PTH) levels, as well as characteristics observed in X-ray imaging indicative of osteopenia or fractures [1,4,7,9,10]. Radiography is not reliable in the early stages of MBD, but radiological alterations can be identified in cases with significant demineralization or fractures [7,10,13]. The gold standard for assessing bone mineral density is dual-energy X-ray absorptiometry [14]. In our hospital, there is no routine monitoring of serum PTH levels and a lack of equipment for dual-energy X-ray absorptiometry for bone mineral density assessment. To diagnose MBD in accordance with the radiological alterations described by Koo’s score [13], we use X-ray imaging interpreted by a single radiologist (to minimize the possibility of bias). The prevalence of MBD in our present study was 21%, showing similarity with previous reports [1,6,7,12].

MBD is a multifactorial disease characterized primarily by inadequate bone matrix mineralization and biochemical alterations in the metabolism of phosphorus and calcium [6,7]. The data in this report indicate that VLBW infants diagnosed with MBD are at an increased risk of neurodevelopmental delay at 2 years of corrected age. Our findings have identified multiple factors associated with an increased risk of MBD, including a lower gestational age, a smaller birth weight, a lower median Apgar score at 5 min after birth, undergoing invasive respiratory support during the fetal–neonatal transition, respiratory distress syndrome, surfactant administration, significant PDA that needed surgical intervention, postnatal steroid use for BPD, and the prolonged use of PN supplements. In agreement with prior reports, a lower gestational age and birth weight, a longer duration of PN, and respiratory distress syndrome with surfactant use were all associated with MBD [7,15,16,17]. Variations in the criteria used to define MBD and in the sample size of each study may account for the differences in the risk factors derived from each report.

Inadequate vitamin D supplementation in the early postnatal period is associated with the development of MBD [17]. In our present study, there was no significant relationship between vitamin D supplementation and the development of MBD. This can be explained by the fact that vitamin D supplements are routinely administered to babies born in our hospital.

Among the risk factors identified in our study, the prolonged use of PN implies a delay in achieving full enteral feeding. It is a direction we can work toward in caring for VLBW infants to avoid MBD. The nutrient and mineral demands of preterm infants are much higher than those of full-term infants [10]. For VLBW infants, PN is limited by the amount of daily fluid intake, resulting in inadequate calcium and phosphorus supplementation [18,19]. Furthermore, the association between a longer duration of PN and neonatal cholestasis has been extensively reported [20,21], and an increased risk of MBD may also result from neonatal cholestasis [16,22,23]. However, its etiology remains unclear.

As the concentration of bile acid decreases, vitamin D and calcium absorption in the intestine may also be affected [22,23]. Toomey et al. demonstrated that the ability to hydroxylate vitamin D3 to 25-hydroxycholecalciferol is impaired in damaged livers (cholestasis with hepatocyte damage), and this might have an impact on the development of MBD [23].

Additionally, it is difficult to identify the causal relationship between these preterm morbidities and MBD because the underlying pathophysiology remains unclear [7]. For example, BPD is defined by a requirement for oxygen supplementation at either 28 days postnatal age or 36 weeks postmenstrual age, and this results in the long-term use of furosemide, methylxanthines, and steroids. These drugs stimulate osteoclast activation, reduce the proliferation of osteoblasts, lower calcium absorption, and increase the renal excretion of calcium [24,25,26], leading to a higher risk of MBD. In contrast, multiple rib fractures from MBD may cause difficulty in ventilator weaning [22]. In our study, morbidities such as cholestasis, BPD, NEC stage ≥ II, culture-proven sepsis, severe ROP, severe IVH, cystic PVL, and post-hemorrhagic hydrocephalus were found to have a linear association with a diagnosis of MBD.

Although MBD resolves on its own in early childhood, the latent long-term consequences demonstrate the importance of preventing the condition [27]. We hypothesized that MBD would affect motor development at 2 years of age because it is highly associated with long bone and rib fractures in early infancy [28]. In our study, we found that infants with MBD had significantly lower BSID-III scores in the cognitive, motor, and language composites at 24 months of corrected age in comparison to those of infants without MBD born with a birth weight of less than 1350 g. Many previous studies have shown that BSID-III scores are higher than BSID-II values, and this implies that clinicians should be aware of the risk of an underestimation of developmental delays based on BSID-III scores [29,30,31]. Yu et al. have asserted that BSID-III composite scores should be adjusted 10–20, 1–13, and 12–24 points higher than 70, respectively, in order to best predict cognitive, language, and motor delays, as a BSID-II index score <70 signifies moderate developmental delay [30]. Celik et al. suggested that cut-off points of 97.5, 92.5, and 98.5 should be used in the BSID-III-based cognitive, language, and motor scores, respectively, for mild developmental delay [31]. While the cut-off values in BSID-III scores for mild, moderate, and severe developmental delays are currently inconclusive, we prefer to use higher scores for a timelier detection of cases with developmental delays, allowing for early intervention. Our study demonstrated that VLBW infants with MBD have a higher risk of mild motor and cognitive delay at 2 years of corrected age. A consistent result was still found after adjusting for confounding factors such as gestational age, cystic PVL, intubation during the fetal–neonatal transition, and cholestasis.

Several risk factors for neurodevelopmental impairment have been discussed in the literature. A systemic review and meta-analysis by Pascal et al. revealed a 16.9% prevalence of cognitive and motor delays in VLBW infants, based on developmental scales at approximately 2 years of corrected age [32]. Lin et al. conducted a population-based prospective cohort study of preterm infants with VLBW born in Taiwan between 2002 and 2009 and showed that lower gestational age, cystic periventricular leukemia, and lower paternal education level had a significant association with disadvantageous neurodevelopmental outcomes [33]. Thanhaeuser et al. reported that infants with PN-associated cholestasis showed significantly lower cognitive, language, and motor scores at both 12 and 24 months of corrected age [34]. These findings are consistent with those of our study.

Overall, our findings highlight the significant association between MBD and neurodevelopmental outcomes that has been insufficiently discussed before. We speculate that an early weaning from PN by shortening the duration to a full oral feeding period may decrease the incidence of MBD in VLBW infants.

A limitation of this study is its retrospective design. The follow-up rate is another factor that limits the power of this study. In most cohort studies, noncompliance with follow-up is a common issue [35]. We have also encountered such problems; however, our study was able to reveal an association between MBD and poor neurodevelopmental outcomes even after controlling for potential confounders. Moreover, for the evaluation of neurodevelopmental impairment, our hospital records lacked data on visual and hearing impairment and cerebral palsy. Nevertheless, we decided to focus on the BSID-III evaluation, considering that MBD is less likely to be related to these diseases.

## 5. Conclusions

This study highlights the association of MBD with poor neurodevelopmental outcomes in cognitive, motor, and language composites at 2 years of corrected age. Further research is required to investigate the attendant mechanisms and inter-relationships.

## Figures and Tables

**Table 1:** Demographics and clinical characteristics of patients.

	No MBD (*n* = 362)	MBD (*n* = 97)	
Characteristics	N (%)	N (%)	*p*-Value
Maternal characteristics			
Age of mother at birth ≥35 y/o	48 (13.3)	20 (20.6)	0.070
Obstetrical condition			
Preeclampsia	75 (20.7)	11 (11.3)	0.036
PPROM	138 (38.1)	45 (46.4)	0.140
Antenatal glucocorticoid			
Any	285 (78.7)	72 (74.2)	0.344
Course completed	244 (67.4)	60 (61.9)	0.305
Type of delivery			0.002
Vaginal	95 (26.2)	41 (42.3)	
Cesarean section	267 (73.8)	56 (57.7)	
Multiple births—no	139 (38.4)	26 (26.8)	0.035
Infant characteristics			
Median gestational age (IQR)—wk	29.1 (27.3–31.6)	25.6 (25.0–27.6)	<0.001
Median birth weight (IQR)—g	1060.5 (895–1211)	742 (635–890)	<0.001
Small for gestational age	156 (43.1)	26 (26.8)	0.004
Male sex	180 (49.7)	43 (44.3)	0.345
Median Apgar score at 5 min (IQR)	8 (7–9)	7 (6–8)	<0.001
Support during fetal–neonatal transition			
Invasive respiratory support	137 (37.8)	78 (80.4)	<0.001
Circulatory support	13 (3.6)	7 (7.2)	0.157
RDS	130 (35.9)	69 (71.1)	<0.001
Surfactant administration	86 (23.8)	48 (49.5)	<0.001
Surgery for PDA	25 (6.9)	47 (48.5)	<0.001
Postnatal steroid for BPD	22 (6.1)	19 (19.6)	<0.001
Use of Vit D3	90 (24.9)	31 (32)	0.159
Median length of PN used (IQR)—d	22.0 (17.0–29.0)	36.0 (28.0–49.0)	<0.001
Median peak serum ALP levels (IQR)—U/L	428.0 (319.0–571.0)	700 (549.0–824.0)	<0.001

*p*-Value calculated using the Mann–Whitney U test or chi-squared test; *p* < 0.05 was deemed statistically significant. MBD, metabolic bone disease; PPROM, preterm premature rupture of membrane; IQR, interquartile ranges; RDS, respiratory distress syndrome; PDA, patent ductus arteriosus; BPD, bronchopulmonary dysplasia; PN, parenteral nutrition; ALP, alkaline phosphatase.

**Table 2:** Comparison of morbidities between infants with and without metabolic bone disease.

	No MBD (*n* = 362)	MBD (*n* = 97)	
Morbidity	N (%)	N (%)	*p*-Value
Cholestasis	25 (6.9)	18 (18.6)	<0.001
BPD	252 (69.6)	95 (97.9)	<0.001
NEC stage ≥II	3 (0.8)	4 (4.1)	0.039
Culture-proven sepsis	53 (14.6)	28 (28.9)	0.001
Severe ROP (stage ≥3)	56 (15.5)	43 (44.3)	<0.001
Severe IVH (≥grade 3)	6 (1.7)	15 (15.5)	<0.001
Cystic PVL	17 (4.7)	13 (13.4)	0.002
Post-hemorrhagic hydrocephalus	1 (0.3)	8 (8.2)	<0.001

*p*-Value by chi-squared test or Fisher’s exact test when appropriate. *p* < 0.05 was deemed statistically significant. MBD, metabolic bone disease; BPD, bronchopulmonary dysplasia; NEC, necrotizing enterocolitis; ROP, retinopathy of prematurity; IVH, intraventricular hemorrhage; PVL, periventricular leukomalacia.

**Table 3:** Bayley Scales among infants with and without metabolic bone disease at 2 years of age.

	No MBD (*n* = 362)	MBD (*n* = 97)	
Bayley-III Scales *	Median (IQR)	Median (IQR)	*p*-Value
Cognitive	90.0 (85.0–95.0)	90.0 (80.0–95.0)	<0.001
Motor	94.0 (88.0–100.0)	88.0 (82.0–94.0)	<0.001
Language	94.0 (86.0–100.0)	89.0 (83.0–97.0)	0.014

*p*-Value by Mann–Whitney U test, *p* < 0.05 was deemed statistically significant. * Bayley Scales of Infant Development-III scores at 24 months of corrected age. IQR, interquartile range; MBD, metabolic bone disease.

**Table 4:** Bayley-III Scales scores among infants presenting with and without fractures diagnosed with metabolic bone disease.

	Osteoporosis Only (*n* = 76)	Osteoporosis with Fracture (*n* = 21)	
Bayley-III Scales *	Median (IQR)	Median (IQR)	*p*-Value
Cognitive	90.0 (80.0–95.0)	90.0 (80.0–95.0)	0.789
Motor	88.0 (82.0–94.0)	88.0 (82.0–94.0)	0.822
Language	90.0 (86.0–97.0)	89.0 (79.0–94.0)	0.712

*p*-Value by Mann–Whitney U test, *p* < 0.05 was deemed statistically significant. * Bayley Scales of Infant Development-III scores at 24 months of corrected age. IQR: interquartile range.

**Table 5:** Multiple generalized linear models * for the Bayley Scales of Infant Development-III.

Outcome	Parameter		Mean Ratio	95% CI	*p*-Value
Cognitive	(Intercept)		86.439	79.061–94.506	<0.001
	Impression rickets or fracture	Yes	0.966	0.943–0.990	0.005
		No	1.000		
	Assessment time	24 months	0.934	0.923–0.946	<0.001
		12 months	0.966	0.954–0.977	<0.001
		6 months	1.000		
	Gestational age		1.004	1.001–1.007	0.005
	Cystic PVL	Yes	0.914	0.869–0.961	<0.001
		No	1.000		
Language	(Intercept)		81.669	74.399–89.649	<0.001
	Impression rickets or fracture	Yes	0.994	0.971–1.017	0.590
		No	1.000		
	Assessment time	24 months	0.994	0.981–1.008	0.422
		12 months	0.968	0.955–0.981	<0.001
		6 months	1.000		
	Gestational age		1.005	1.001–1.008	0.004
	Cystic PVL	Yes	0.949	0.906–0.994	0.028
		No	1.000		
	Steroids for chronic lung disease	Yes	0.947	0.914–0.981	0.002
		No	1.000		
Motor	(Intercept)		93.563	92.091–95.059	<0.001
	Impression rickets or fracture	Yes	0.951	0.922–0.982	0.002
		No	1.000		
	Assessment time	24 months	1.033	1.018–1.047	<0.001
		12 months	1.045	1.031–1.060	<0.001
		6 months	1.000		
	Intubation during fetal–neonatal transition	Yes	0.956	0.936–0.977	<0.001
		No	1.000		
	Cystic PVL	Yes	0.870	0.815–0.930	<0.001
		No	1.000		
	Cholestasis	Yes	0.939	0.898–0.982	0.005
		No	1.000		

*p* < 0.05 was deemed statistically significant. * Multiple generalized linear models with log link and gamma distribution in the GEE method. GEE, generalized estimating equation; PVL, periventricular leukomalacia; CI, confidence interval.

## Data Availability

All data relevant to the study are included in the article.

## References

[1] Faienza M.F., D’Amato E., Natale M.P., Grano M., Chiarito M., Brunetti G., D’Amato G. (2019). Metabolic bone disease of prematurity: Diagnosis and management. Front. Pediatr..

[2] Abrams S.A., Committee on Nutrition (2013). Calcium and vitamin D requirements of enterally fed preterm infants. Pediatrics.

[3] Backström M.C., Kuusela A.L., Mäki R. (1996). Metabolic bone disease of prematurity. Ann. Med..

[4] Vachharajani A.J., Mathur A.M., Rao R. (2009). Metabolic bone disease of prematurity. NeoReviews.

[5] Harrison C.M., Johnson K., McKechnie E. (2008). Osteopenia of prematurity: A national survey and review of practice. Acta Paediatr..

[6] Perrone M., Casirati A., Stagi S., Amato O., Piemontese P., Liotto N., Orsi A., Menis C., Pesenti N., Tabasso C. (2022). Don’t forget the bones: Incidence and risk factors of metabolic bone disease in a cohort of preterm infants. Int. J. Mol. Sci..

[7] Avila-Alvarez A., Urisarri A., Fuentes-Carballal J., Mandiá N., Sucasas-Alonso A., Couce M.L. (2020). Metabolic bone disease of prematurity: Risk factors and associated short-term outcomes. Nutrients.

[8] Neu J., Walker W.A. (2011). Necrotizing enterocolitis. N. Engl. J. Med..

[9] Visser F., Sprij A.J., Brus F. (2012). The validity of biochemical markers in metabolic bone disease in preterm infants: A systematic review. Acta Paediatr..

[10] Chinoy A., Mughal M.Z., Padidela R. (2019). Metabolic bone disease of prematurity: Causes, recognition, prevention, treatment and long-term consequences. Arch. Dis. Child. Fetal Neonatal Ed..

[11] Motte-Signoret E., Jlassi M., Lecoq L., Wachter P.Y., Durandy A., Boileau P. (2023). Early elevated alkaline phosphatase as a surrogate biomarker of ongoing metabolic bone disease of prematurity. Eur. J. Pediatr..

[12] Mitchell S.M., Rogers S.P., Hicks P.D., Hawthorne K.M., Parker B.R., Abrams S.A. (2009). High frequencies of elevated alkaline phosphatase activity and rickets exist in extremely low birth weight infants despite current nutritional support. BMC Pediatr..

[13] Koo W.W., Gupta J.M., Nayanar V.V., Wilkinson M., Posen S. (1982). Skeletal changes in preterm infants. Arch. Dis. Child..

[14] Gharibeh N., Razaghi M., Vanstone C.A., Sotunde O.F., Glenn L., Mullahoo K., Farahnak Z., Khamessan A., Wei S.Q., McNally D. (2023). Effect of vitamin D supplementation on bone mass in infants with 25-hydroxyvitamin D concentrations less than 50 nmol/L: A prespecified secondary analysis of a randomized clinical trial. JAMA Pediatr..

[15] Viswanathan S., Khasawneh W., McNelis K., Dykstra C., Amstadt R., Super D.M., Groh-Wargo S., Kumar D. (2014). Metabolic bone disease: A continued challenge in extremely low birth weight infants. JPEN J. Parenter. Enteral Nutr..

[16] Ukarapong S., Venkatarayappa S.K.B., Navarrete C., Berkovitz G. (2017). Risk factors of metabolic bone disease of prematurity. Early Hum. Dev..

[17] Chen W., Yang C., Chen H., Zhang B. (2018). Risk factors analysis and prevention of metabolic bone disease of prematurity. Medicine.

[18] Hekimoğlu B.S. (2023). Risk factors and clinical features of osteopenia of prematurity: Single-center experience. Trends Pediatr..

[19] Ferrone M., Geraci M. (2007). A review of the relationship between parenteral nutrition and metabolic bone disease. Nutr. Clin. Pract..

[20] Alkharfy T.M., Ba-Abbad R., Hadi A., Sobaih B.H., AlFaleh K.M. (2014). Total parenteral nutrition-associated cholestasis and risk factors in preterm infants. Saudi J. Gastroenterol..

[21] Jolin-Dahel K., Ferretti E., Montiveros C., Grenon R., Barrowman N., Jimenez-Rivera C. (2013). Parenteral nutrition-induced cholestasis in neonates: Where does the problem lie?. Gastroenterol. Res. Pract..

[22] Dabezies E.J., Warren P.D. (1997). Fractures in very low birth weight infants with rickets. Clin. Orthop. Relat. Res..

[23] Toomey F., Hoag R., Batton D., Vain N. (1982). Rickets associated with cholestasis and parenteral nutrition in premature infants. Radiology.

[24] Bozzetti V., Tagliabue P. (2009). Metabolic bone disease in preterm newborn: An update on nutritional issues. Ital. J. Pediatr..

[25] Saarela T., Vaarala A., Lanning P., Koivisto M. (1999). Incidence, ultrasonic patterns and resolution of nephrocalcinosis in very low birthweight infants. Acta Paediatr..

[26] Hoppe B., Duran I., Martin A., Kribs A., Benz-Bohm G., Michalk D.V., Roth B. (2002). Nephrocalcinosis in preterm infants: A single center experience. Pediatr. Nephrol..

[27] Fewtrell M.S., Cole T.J., Bishop N.J., Lucas A. (2000). Neonatal factors predicting childhood height in preterm infants: Evidence for a persisting effect of early metabolic bone disease?. J. Pediatr..

[28] Högberg U., Andersson J., Högberg G., Thiblin I. (2018). Metabolic bone disease risk factors strongly contributing to long bone and rib fractures during early infancy: A population register study. PLoS ONE.

[29] Rogers E.E., Hintz S.R. (2016). Early neurodevelopmental outcomes of extremely preterm infants. Semin. Perinatol..

[30] Yu Y.-T., Hsieh W.-S., Hsu C.-H., Chen L.-C., Lee W.-T., Chiu N.-C., Wu Y.-C., Jeng S.-F. (2013). A psychometric study of the Bayley Scales of Infant and Toddler Development–3rd Edition for term and preterm Taiwanese infants. Res. Dev. Disabil..

[31] Çelik P., Ayranci Sucakli I., Yakut H.I. (2020). Which Bayley-III cut-off values should be used in different developmental levels?. Turk. J. Med. Sci..

[32] Pascal A., Govaert P., Oostra A., Naulaers G., Ortibus E., Van den Broeck C. (2018). Neurodevelopmental outcome in very preterm and very-low-birthweight infants born over the past decade: A meta-analytic review. Dev. Med. Child. Neurol..

[33] Lin C.Y., Hsu C.H., Chang J.H., Taiwan Premature Infant Follow-Up Network (2020). Neurodevelopmental outcomes at 2 and 5 years of age in very-low-birth-weight preterm infants born between 2002 and 2009: A prospective cohort study in Taiwan. Pediatr Neonatol.

[34] Thanhaeuser M., Steyrl D., Fuiko R., Brandstaetter S., Binder C., Thajer A., Huber-Dangl M., Haiden N., Berger A., Repa A. (2022). Neurodevelopmental outcome of extremely low birth weight infants with cholestasis at 12 and 24 months. Neonatology.

[35] Kim N.H., Youn Y.A., Cho S.J., Hwang J.-H., Kim E.-K., Kim E.A.-R., Lee S.M., Network K.N. (2018). The predictors for the non-compliance to follow-up among very low birth weight infants in the Korean neonatal network. PLoS ONE.

